# The Biogenesis, Biological Functions, and Applications of Macrophage-Derived Exosomes

**DOI:** 10.3389/fmolb.2021.715461

**Published:** 2021-07-21

**Authors:** Xiaoxiao Shan, Caiyun Zhang, Chutian Mai, Xuerui Hu, Nuo Cheng, Weidong Chen, Daiyin Peng, Lei Wang, Zhaojie Ji, Ying Xie

**Affiliations:** ^1^ School of Pharmacy, Anhui Academy of Chinese Medicine, , Anhui University of Traditional Chinese MedicineHefei, China; ^2^ Anhui Province Key Laboratory of Chinese Medicinal Formula, Hefei, China; ^3^ Anhui Province Key Laboratory of Pharmaceutical Technology and Application, Hefei, China; ^4^ State Key Laboratory of Quality Research in Chinese Medicines, Macau University of Science and Technology, Avenida Wai Long, China

**Keywords:** macrophage-derived exosomes, formation mechanisms, polarization, biological functions, applications

## Abstract

Macrophage-derived exosomes have been implicated on the modulation of inflammatory processes. Recent studies have shown that macrophage-derived exosomes contribute to the progression of many diseases such as cancer, atherosclerosis, diabetes and heart failure. This review describes the biogenesis of macrophage-derived exosomes and their biological functions in different diseases. In addition, the challenges facing the use of macrophage-derived exosomes as delivery tools for drugs, genes, and proteins in clinical applications are described. The application of macrophage-derived exosomes in the diagnosis and treatment of diseases is also discussed.

## Introduction

Exosomes are lipid bilayer particles that are actively secreted out of the cell. Pan et al. reported a small vesicle from the supernatant of sheep reticulocytes, which was initially thought to be a cell-secreted waste product (Pan et al., 1985). Further studies on exosomes reported that vesicles comprise several components including cell-specific proteins, lipids and RNA (mRNA, miRNA and other non-coding RNA) (Llorente et al., 2017; Tomasetti et al., 2017). Exosomes can be secreted by a variety of cells, for example, in 1996, Raposo et al. reported that B lymphocytes secrete antigen-presenting vesicles (Raposo et al., 1996). Notably, exosomes secreted by immune cells such as dendritic cells (DCs) modulate the immune response, therefore, these membranous vesicles are being explored as potential immunotherapeutic reagents (Greening et al., 2015). Natural killer (NK) cells exhibit rapid immunity to metastatic or hematological malignancies, and clinical studies are being conducted to explore the antitumor properties of NK cells. A study by Zhu et al. reported that exosomes derived from NK cells (NK-Exos) exert cytotoxic effects on melanoma cells (Zhu et al., 2017). Mast cells are important effector cells of the immune system. Mast cell-derived exosomes carrying RNAs play a role in immune regulation (Liang et al., 2018). Exosomes are widely distributed in various body fluids including blood, urine, peritoneal fluid, synovial fluid and breast milk. They affect the physiological and pathological state of the target cells by carrying and transmitting important signaling molecules to these cells. Extracellular vesicles are grouped into three main categories based on size, biological properties, and formation process. These include exosomes (30–150 nm), microvesicles (200–1,000 nm), and apoptotic bodies (500–2000 nm) (Shao et al., 2018). Exosomes are formed by the intranuclear body system. Formation, sorting of the encapsulated contents, and release of exosomes are regulated by a series of precise regulatory mechanisms. Microvesicles are formed by outgrowth of the cell membrane. Formation is mainly induced stimulation of the redistribution of the phospholipid bilayer from the cell membrane by inward flow of Ca^2+^, leading to outgrowth of the cell membrane (Akers et al., 2013). However, the molecular mechanism of microvesicle formation has not been fully elucidated. Apoptotic bodies are formed when the cell membrane crumples, and invaginates during apoptosis, shedding organelles, and nuclear debris with wrapped cytoplasm (Gurunathan et al., 2019). Exosomes are the smallest extracellular vesicles and play key biological roles (Kalluri and LeBleu, 2020). Therefore, exosomes have been explored as novel potential therapeutic tools owing to their ability to modulate various biological processes, including immune response, cell proliferation, cell invasiveness, synapsis plasticity, angiogenesis and tubule formation. Moreover, high levels of macrophage-derived exosomes in blood makes them potential biomarkers for minimally invasive liquid biopsies for diagnosis and prognosis in cancer patients (Ismail et al., 2013; Jin et al., 2015; Lin and Dihua, 2019).

Macrophages are multifunctional cell types presenting in most vertebrate tissues. They form the first line of defense against pathogens through phagocytosis of microbial infections, particles and dead cells (Verdeguer and Aouadi, 2017). Macrophages are heterogeneous cells, and the phenotypes and functions are regulated by the surrounding microenvironment (Shapouri-Moghaddam et al., 2018; Yang et al., 2019b). Macrophages are classified into classically activated (M1) and alternatively activated (M2) macrophages based on whether they mediate anti-inflammatory or pro-inflammatory responses (Olefsky and Glass, 2010; Murray et al., 2014; Verdeguer and Aouadi, 2017). Metabolites associated with microbial infections, such as lipopolysaccharide (LPS) and interferon-gamma (INF-γ), induce secretion of inflammatory factors by macrophages, such as tumor necrosis factor-alpha (TNF-α) and interleukin-12 (IL-12). Therefore, they stimulate the body's immune response by triggering a typical pro-inflammatory response. Notably, macrophages are polarized to M2a in response to IL-4 or IL-13, M2b in response to immune complexes and M2c in response to the anti-inflammatory cytokine, interleukin-10 (IL-10). This polarization induces macrophages to secrete anti-inflammatory factors, such as Arginase-1 (Arg-1) and transforming growth factor-β (TGF-β), thus reducing inflammatory response and promoting wound healing (Atri et al., 2018; Funes et al., 2018). Polarization of macrophages is implicated in development and progression of several diseases and study on macrophages enables understanding of macrophage-derived exosomes (Lee et al., 2014). Exosomes carry biological information on macrophages and play an important regulatory role in several diseases, such as tumors, inflammations, and atherosclerosis (Théry et al., 2002). Macrophage-derived exosomes are more than exosomes from other cell sources, and are biocompatible thus they can be used as drug carriers for drug delivery (Kim et al., 2018). In the current review, the mechanisms of formation, classification, and function of macrophage-derived exosomes were explored. In addition, application of macrophage-derived exosomes as delivery tools of drugs, genes, and proteins was reviewed. Deeper understanding of macrophage-derived exosomes may provide possible therapeutic targets for various diseases.

## Formation Mechanisms of Macrophage-Derived Exosomes

**FIGURE 1:**
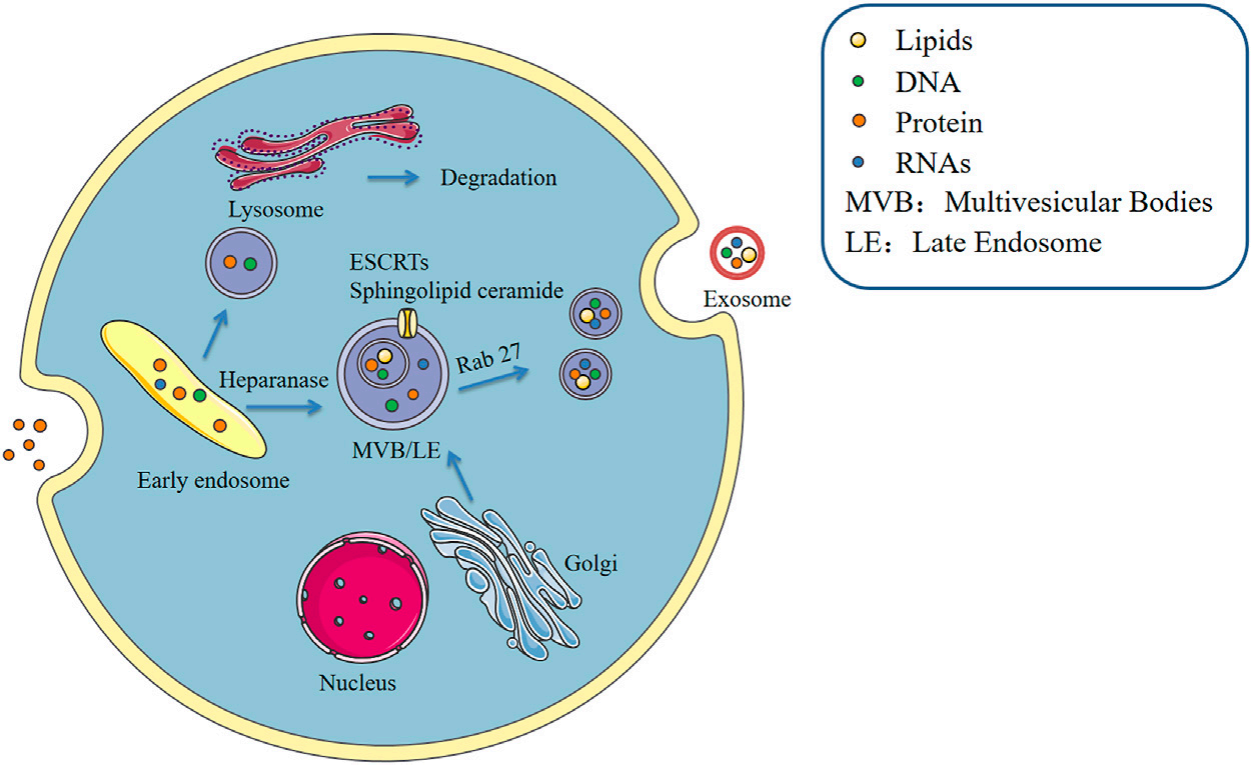
Formation of exosomes.

Formation of macrophage-derived exosomes, similar to that of most cell-derived exosomes, takes place in three main stages including exosome biogenesis, sorting of cargo into exosome and exosome release (Kalluri and LeBleu, 2020). This process is precisely regulated and involving multiple proteins. The cytoplasmic membrane of the macrophage initially invaginates to form endocytic vesicles, and multiple endocytic vesicles fuse to form early endosomes. The early endosomes then invaginate, encapsulating intracellular material in the process, thus forming multiple intracellular vesicles (ILVs) and further transforming into late endosomes, which are known as multivesicular bodies (MVBs). MVBs then fuse with the cytoplasmic membrane and release ILVs into the extracellular space as exosomes. ESCRT (endosomal sorting complex required for transport) pathway is the most explored mechanism for ILV and MVB formation. ESCRT machinery comprises four multimeric complexes and associated proteins that assemble in an ordered manner at the endosome (Friand et al., 2015). ESCRTs comprise approximately twenty proteins that assemble into four complexes (ESCRT-0, -I, -II, and -III) with associated proteins including VPS4, VTA1 and ALIX (Colombo et al., 2013). ESCRT-0, -I, and -II complexes recognize and sequester ubiquitinated membrane proteins at the endosomal delimiting membrane, whereas ESCRT-III complex plays a role in membrane budding and actual scission of ILVs (Raposo and Stoorvogel, 2013). In addition to controlling exosome unharness, ESCRTs are implicated in packaging of biomolecules into exosomes. (Pipe and Katzmann, 2006). Heparanase is a modulator of the syndecan-syntenin-ALIX pathway that induces endosomal membrane budding. This leads to formation of exosomes by trimming the heparan sulphate chains on syndecans (Roucourt et al., 2015). However, studies report that the cargo is segregated into distinct subdomains on the endosomal membrane (Hunt et al., 2013). In addition, transfer of exosome-associated domains into the lumen of the endosome does not depend on the function of the ESCRT, however, it is induced by sphingolipid ceramide. Purified exosomes comprise ceramide, and release of exosomes is reduced by inhibition of neutral sphingomyelinases (Trajkovic et al., 2008). This implies that in addition to proteins, lipids play a regulatory role during release of exosomes. Furthermore, protein sorting in MVBs is mediated by ESCRT-dependent and independent pathways (Figure 1).

Macrophage secretion of exosomes is stimulated by the extracellular environment. During the process of cargo sorting, the macrophage P2X7 signaling pathway is activated through stimulation of extracellular adenosine triphosphate (ATP) and the intracellular calcium concentration increases, promoting entry of proteins such as IL-1β into exosomes (Qu et al., 2007). Rab GTPases, the largest family of small GTPases, regulate several steps of membrane trafficking, including vesicle budding, transport of vesicles along actin and tubulin, and membrane fusion (Stenmark, 2009). LPS induces the release process of macrophage exosomes, however, this effect can be reversed by IL-25, which also downregulates expression of Rab27a and Rab27b in macrophages. IL-25 thus inhibits exosome release (Li et al., 2018). Pretreatment of RAW264.7 macrophages with the exosome secretion inhibitor GW4869, followed by stimulation with LPS, causes a reduction in macrophage exosome secretion and reduction in inflammatory factor secretion. Therefore, blocking exosome production in sepsis can inhibit sepsis-induced inflammatory response thus improving cardiac function and survival (Essandoh et al., 2015). In addition, most mature MVB are broken down by lysosomes and the products are released into the extracellular environment as extracellular bodies with the help of Rab proteins and small GTPases by cytosolic spitting. This indicates that the function of lysosomes is closely linked to secretion of exosomes. In alcoholic liver disease, alcohol decreases lysosomal function in hepatocytes and macrophages, and expression of lysosome-associated membrane protein 1 (LAMP1) and LAMP2 is downregulated. Amphisome in macrophages does not bind to lysosomes, resulting in increase in the level of exosome release (Liu et al., 2020a). These findings have significant implications in understanding the role of different cell types and different cellular environments in modulating exosome release. Increase or decrease in secretion of macrophage-derived exosomes has different implications for different diseases.

## Different Phenotypes of Macrophage-Derived Exosomes

### Macrophage Metabolism and Polarization

**FIGURE 2:**
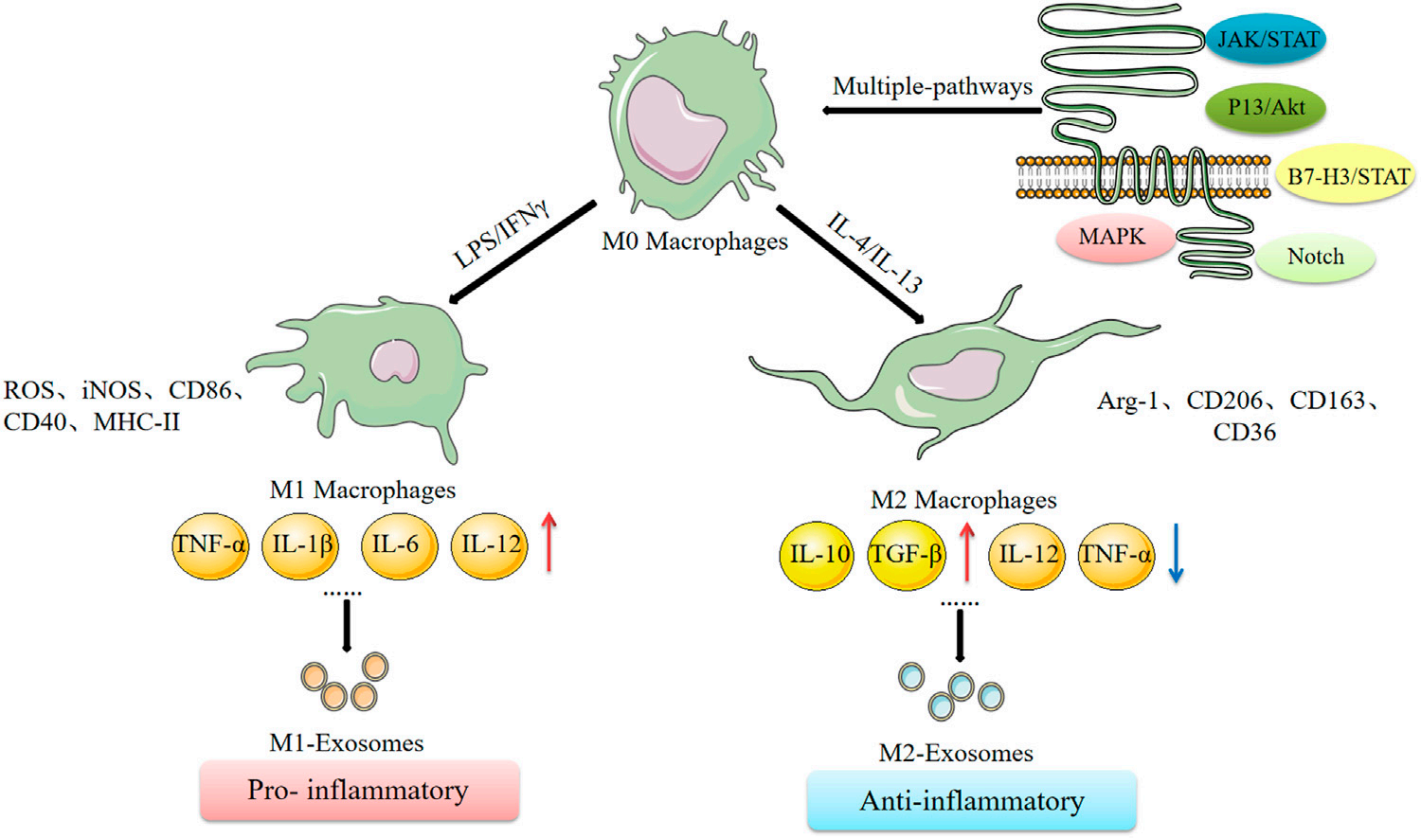
Macrophage polarization and its related mechanisms.

Macrophages are heterogeneous and their phenotype and functions are regulated by the surrounding micro-environment. An IL-4-mediated macrophage phenotype known as alternatively activated (M2) macrophages was reported in the early 1990s. This phenotype was characterized by high clearance of mannosylated ligands, enhanced expression of MHC II antigens, and reduced secretion of pro-inflammatory cytokines compared with the classically activated M1 macrophages induced by IFN-γ (Martinez and Siamon, 2014). This classification is based on the phenotypic changes observed *in vitro* after stimulation by different cytokines (Stein et al., 1992). Different typologies of macrophages and their cell expression markers are presented in Figure 2. Reprogramming of intracellular metabolism is necessary for effective polarization and function of activated macrophages. M1 macrophages increase glucose consumption and lactate release, whereas M2 macrophages predominantly promote the oxidative glucose metabolic pathway. In the tumor microenvironment, glucose metabolism of tumor-associated macrophages (TAMs) mainly takes place through aerobic glycolysis. Inhibition of aerobic glycolysis of TAMs can convert the tumor-promoting M2-TAMs to the tumor-inhibiting M1-TAMs, thus inhibiting tumor development (Linnan et al., 2015). Mills et al. reported that oxidized succinate and mitochondrial membrane potential in the mitochondria of macrophages is increased by LPS stimulation, and succinate dehydrogenase (SDH) promotes mitochondrial reactive oxygen species (ROS) production (Mills et al., 2016). This implies that the macrophage function shifted from oxidative phosphorylation to ATP production to glycolysis, resulting in increased succinate levels. Moreover, succinate promotes LPS-induced glycolysis in macrophages and promotes and maintains expression of endogenous pro-inflammatory genes and inhibits expression of anti-inflammatory genes (O'Neill and Pearce, 2015). The carbohydrate kinase-like protein (CARKL) induces macrophage polarization by regulating glucose metabolism (Haschemi et al., 2012). Succinic acid regulates the pro-inflammatory IL-1β-HIF-1α axis, whereas itaconate exerts anti-inflammatory effects by inhibiting succinate dehydrogenase-mediated oxidation of succinate thus regulate macrophage metabolism (Lampropoulou et al., 2016). These studies report an interactive relationship between metabolic reprogramming and macrophage polarization. Understanding the relationship between cellular metabolism and macrophage polarization provides an insight into the molecular mechanisms underlying functions of exosomes in cancer development.

### Differences Between Macrophage-Derived Exosome Subtypes

Three types of macrophage-derived exosomes including unpolarised M0 macrophage-derived exosomes (M0-Exos), polarised M1 and M2 macrophage-derived exosomes (M1-Exos and M2-Exos) have been explored (Funes et al., 2018). Variations between exosomes derived from completely different phenotypes of macrophages have been reported which reflect the parental cell properties. For example, M2-Exos contain more miR-365 than M1-Exos, and blocking this miRNA restores sensitivity of cancer cells to gemcitabine (Binenbaum et al., 2018). M1-Exos have high levels of miR-326 and suppress proliferation, migration, and invasion, and promote apoptosis of hepatocellular carcinoma cells (HCC), through downregulation of NF-κB expression in HCC by miR-326 (Bai et al., 2020). Long non-coding RNAs (lncRNAs) play key roles in multiple diseases. Wu et al. reported that the lncRNA PVT1 carried by M2-Exos acts as a miR-21-5p sponge to upregulate the cytokine signaling repressor protein, SOCS5 and inactivates the JAKs/STAT3 pathway (Wu et al., 2020b). M1-Exos containing miR-16-5p inhibit gastric cancer progression by activating T-cell immune responses through PD-L1 (Li et al., 2020b). Furthermore, M0 macrophage-derived extracellular transfer of miR-223 induces resistance to adriamycin in gastric cancer (Gao et al., 2020). In summary, these findings indicate that different phenotypes of macrophage-derived exosomes contain different biological information, thus perform different functions. Several studies on exosomes have nit explored whether macrophages are polarized or not and the origin of macrophage exosomes is not fully elucidated in these studies. Macrophage-derived exosomes increases MMP-2 expression in vascular smooth muscle cells through activation of JNK and p38 pathways, thus promoting abdominal aortic aneurysm (Wang et al., 2019a). Macrophage-derived exosomes can directly inhibit pro-inflammatory enzymes and cytokines such as IL-6 and TNF-α in diabetic wound dysfunction to achieve anti-inflammatory effects and further induce endothelial cell proliferation and migration to accelerate the wound healing process (Li et al., 2019b). Exosomes are “nanospheres”, which contain proteins and lipids from parental cells, mainly including tetraspanin (CD9, CD63 and CD81), proteins involved in biosynthesis of multivesicles (such as Alix and TSG101), heat shock proteins (HSP70 and HSP90) and membrane translocation and fusion proteins (GTPases and membrane coupling proteins). These protein markers of exosomes were originally identified by mass spectrometry during purification of exosomes as highly abundant proteins present in extracellular vesicles, thus these proteins were used as markers of extracellular vesicles. However, currently there are no biomarkers that can distinguish M1-Exos from M2-Exos (Table 1). Application of new technologies, such as neighborhood coding techniques (Wu et al., 2019), may facilitate differentiation of exosomes from different origins.

**TABLE 1:** Biomarkers of macrophage-derived exosomes.

Protein type	Protein name	References
Tetraspanin	CD9, CD63, CD81	(Jin et al., 2015; Deng et al., 2018)
ESCRT proteins	Alix, TSG101	(Diaz et al., 2018; Jeppesen et al., 2019)
Heat shock proteins	HSP60, HSP70, HSPA5, CCT2, HSP90	(Mathivanan et al., 2010; Deng et al., 2018)
Membrane translocation and fusion proteins	GTPases, membrane coupling proteins, annexins, flotillin	(Théry et al., 2002; Liu et al., 2017)

## The Biological Functions of Macrophage-Derived Exosomes

### Role of Macrophage-Derived Exosomes in the Tumor Microenvironment

Tumor microenvironment is a local homeostatic environment comprising tumor cells, macrophages, fibroblasts, and extracellular matrix. It plays an important role in development, recurrence, metastasis, and chemotherapy resistance of cancer (Perrin et al., 2019; Vitale et al., 2019). Macrophage-derived exosomes are one of the independent components of the tumor microenvironment once they are released into the extracellular environment and they play their functions in the tumor microenvironment (Liu et al., 2020b). Tumor-associated macrophages (TAMs) are similar to M2-polarized macrophages, which are activated by Th2 cytokines (IL-4, IL-10, and IL-13) (Lan et al., 2019). MiR-501-3p in M2-Exos promotes tumor development by activating the transforming growth factor-β signaling pathway and inhibiting the tumor suppressor gene TGFBR3 (Yin et al., 2019). M2-Exos transfer LncRNA AFAP1-AS1, down-regulate miR-26a and up-regulate activating transcription factor 2 (ATF2), thus promoting esophageal cancer invasion and metastasis. Targeting M2 macrophages and the lncRNA AFAP1AS1/miR-26a/ATF2 signaling axis is a potential therapeutic strategy for esophageal cancer (Mi et al., 2020). Apolipoprotein E (ApoE) is a highly specific protein in M2-Exos. M2-Exos mediate intercellular transfer of the ApoE-activated PI3K-Akt signaling pathway within recipient gastric cancer cells thus it can remodel cytoskeleton-supporting migration (Zheng et al., 2018). Similarly, Lan et al. reported that M2-Exos exhibit a regulatory effect on BRG1 through delivery of miR-21 and miR-155-5p, thus downregulating BRG1 to promote colorectal cancer metastasis (Lan et al., 2019). miRNAs carried by M2-Exos are important targets for reversing tumor migration whereas altering the phenotype of macrophages can be used to regulate the tumor microenvironment. Exosomes derived from M1 macrophages repolarize M2 macrophages into M1 macrophages, thus they are used to enhance anti-cancer effects of immune checkpoint inhibitors such as aPD-L1 (Choo et al., 2018). Notably, study reports that M1-Exos can polarize macrophages into M1 macrophages. M1-Exos activate the macrophage NF-κB pathway through a caspase-3-mediated pathway, promoting release of inflammatory cytokines, thus establishing a local inflammatory environment and enhancing their anti-tumor activity (Wang et al., 2019b).

### Role of Macrophage-Derived Exosomes in Atherosclerosis

Macrophage-derived exosome-mediated cell-cell communication plays an important role in atherosclerotic processes. Oxidized low-density lipoprotein (ox-LDL) promotes dysregulation of the metabolism of lipoproteins and deposition of lipoproteins in the arterial wall. In addition, ox-LDL is implicated in initiation and development of atherosclerosis (AS). ox-LDL stimulates macrophage-derived exosomes and mediates endothelial cell growth and tube-forming capacity. Notably, blocking exosome secretion rescues endothelial cell growth and tube-forming capacity (Huang et al., 2018). Nguyen et al. reported that the expression profile of ox-LDL stimulated macrophage-derived exosomal miRNAs and exosomal miRNAs, mainly miR-146a, may accelerate development of atherosclerosis by reducing cell migration and promoting macrophage capture in the vessel wall (Nguyen et al., 2018). Further studies report that miR-146a is enriched in serum-derived exosomes from atherosclerotic patients and ox-LDL-treated macrophage-derived exosomes. Exosomal miR-146a secreted by ox-LDL-treated macrophages accelerates AS by targeting superoxide dismutase 2 (SOD2) and promoting release of reactive oxygen species (ROS) and neutrophil extracellular traps (NETs) (Zhang et al., 2019). Moreover, increased expression of macrophage-derived exosomal miRNA-21-3p exhibits similar activities to those of miR-146a (Zhu. et al., 2019). MSC-derived exosomes attenuate atherosclerotic progression through miR-let7-mediated infiltration and polarization of M2 macrophages (Li et al., 2019a). Wu et al. electroporated M2-Exos with hexyl 5-aminolevulinate hydrochloride (HAL) (Wu et al., 2020a). After systemic administration, the molecularly engineered M2-Exos exhibited good chemotactic and anti-inflammatory effects, which promoted release anti-inflammatory cytokines from anti-inflammatory M2 macrophages by binding to surface-bound chemokine receptors. Furthermore, encapsulated HAL can produce anti-inflammatory carbon monoxide and bilirubin through endogenous biosynthesis and metabolism of hemoglobin, thus further promoting anti-inflammatory effect and ultimately reducing AS. Although the role of macrophage-derived exosomes in atherosclerosis has received mixed reviews, exosomal miRNA and lncRNA are more stable compared with serum RNA, and altering their levels in exosomes may be more valuable in treatment of AS.

### Role of Macrophage-Derived Exosomes in Diabetes

Obesity is a risk factor for diabetes and is correlated with intracellular stress, low-grade inflammation, over-activation of the inflammatory response, and imbalance in M1-M2 polarization of macrophages (Bouloumié et al., 2005). A previous study reports that adipose tissue macrophage (ATM) secreted exosomes from obese mice cause abnormal glucose tolerance and insulin resistance when administered to lean mice. On the contrary, ATM exosomes harvested from lean mice improved glucose tolerance and insulin sensitivity when administered to obese recipients. Further, *in vivo* and *in vitro* studies of ATM-secreted exosomes report that miRNAs in the exogenous genes cause modulate insulin signaling (Ying et al., 2017). Exosomes secreted by macrophages exhibit no effect on differentiation from preadipocytes to adipocytes, fat storage, and insulin-mediated glucose uptake. However, miRNAs in LPS-activated macrophage exosomes are highly variable, for instance miR-530, chr16_34840, and chr9_22532 are highly expressed in these exosomes (De et al., 2018). miRNA-mediated pathogenesis of diabetes contained in macrophage-derived exosomes can be explored as a new target for development of diabetes diagnosis approaches and clinical therapy. Tian et al. reported that miR-210 in adipose tissue macrophages regulates glucose uptake and mitochondrial complex IV (CIV) activity by targeting ubiquinone 1 alpha subcomplex 4 (NDUFA4) gene expression, thus promoting development of obesity diabetes in mice (Tian et al., 2020). Diabetic foot disease is a major complication of diabetes. Macrophage-derived exosomes significantly reduce secretion of pro-inflammatory cytokines and promote proliferation and migration of endothelial cells, thus improving angiogenesis and re-epithelialization of diabetic wounds (Li et al., 2019b). However, the study did not elucidate the type of macrophages implicated in this role (Li et al., 2019b). Furthermore, a previous study reported that M2 macrophages improve high glucose (HG)-induced podocyte apoptosis and epithelial-mesenchymal transition by secreting miR25-3p in exosomes and confirmed that dual-specificity protein phosphatase 1 (DUSP1) was the downstream target. MiR25-3p acts by inhibiting DUSP1 expression to activate cellular autophagy (Huang et al., 2020). In summary, the ability of macrophage-derived exosomes to modulate the inflammatory microenvironment and to express miRNAs implies that it is a potential therapeutic strategy for treatment of diabetes-related metabolic diseases.

### Role of Macrophage-Derived Exosomes in Heart Disease

A fine-tuned balance between M1 and M2 macrophage states is important for myocardial repair. Although M1 macrophages play a key role in the immune response of the heart, they promote pro-inflammatory state and degradation of extracellular matrix and cell death. Stimulation of macrophage polarization towards M2 phenotype promotes regression of inflammation and facilitates infarct healing after acute myocardial infarction (Zhou et al., 2015). M2-Exos carrying miR-148a alleviates myocardial ischemia/reperfusion (MI/R) injury by down-regulating thioredoxin-interacting protein (TXNIP) and through inactivation of the TLR4/NF-κB/NLRP3 inflammasome signaling pathway (Dai et al., 2020). MiR-155 is a specific marker for M1 macrophage differentiation and a mediator of miRNA, and is one of the most abundant miRNAs in M1-Exos (Jablonski et al., 2016). Recent studies explored the role of miR-155 in myocardial injury. Wang et al. reported high expression levels of miR-155 in exosomes of activated macrophages (Wang et al., 2017). Notably, miR-155-enriched exosomes suppressed fibroblast proliferation and promoted fibroblast inflammation (Wang et al., 2017). Furthermore, miR-155 downregulation significantly decreased incidence of cardiac rupture and improved cardiac function after acute myocardial infarction (AMI) (Wang et al., 2017). However, the study did not explore whether the stimulated macrophages were M1 macrophages. A previous study reported that M1-Exos inhibit Sirt1/AMPKα2 endothelial nitric oxide synthase and RAC1-PAK2 signaling pathways by targeting five molecular nodes (genes) through delivery of miR-155 to endothelial cells. These events reduce the angiogenic capacity of endothelial cells, exacerbate myocardial injury and inhibit cardiac healing (Liu et al., 2020b). Fusion of released exosomes with the plasma membrane results in release of miR-155 into the cytosol and translational repression of forkhead transcription factors of the O class (FoxO3a) in cardiomyocytes. Macrophage-derived miR-155-containing exosomes promote cardiomyocyte pyroptosis and uremic cardiomyopathy changes by directly targeting FoxO3a in uremic mice (Wang et al., 2020). These findings indicate that inhibition of secretion of miR-155-containing macrophage-derived exosomes, or targeted inhibition of miR-155 gene expression is a novel strategy for treatment of cardiomyopathies.

### Role of Macrophage-Derived Exosomes in Inflammation

Macrophage-derived exosomes are highly correlated with inflammation. A previous study explored the effects of different types of M2 macrophage-derived exosomes (M2a, M2b, and M2c macrophage-derived exosomes) on inflammatory bowel disease (IBD) induced by dextran sodium sulfate (DSS). The findings showed that although all types of M2 macrophage-derived exosomes reduced severity of IBD, M2b macrophage-derived exosomes were more effective compared with M2a and M2c macrophage-derived exosomes. M2b macrophage-derived exosomes carry Chemokine (C-C Motif) Ligand 1 (CCL1) protein to the colon, which interacts with its ligand C-C chemokine receptor 8 (CCR8), and promotes Th2 cells polarization, thus increases levels of Treg cells and reduces production of pro-inflammatory cytokines in the colon (IL-1β, IL-6, and IL-17A) (Yang et al., 2019a). Diabetic wound dysfunction is a severe, chronic complication of diabetes, and is characterized by continuous inflammatory response leading to impaired wound healing. Li et al. reported that exosomes inhibited activation of the AKT (P-AKT) signaling pathway, down-regulated expression of MMP-9, reduced secretion of inflammatory factors, improved pathophysiological status of diabetic wounds, and accelerated healing process in diabetic rats after administration of macrophage-derived exosomes Li et al., 2019b). Moreover, MSCs can release several exosomes with superior regulatory and regenerative abilities thus maintaining the balance of macrophages and improving the resolution of chronic inflammation after LPS treatment. LPS pretreated MSC-derived exosomes promote conversion of macrophages to an M2-like phenotype by shuttling let-7b (Ti et al., 2015). Macrophages infiltrate blood vessels and release exosomes that interact with endothelial cells during hypertension thus increasing inflammation by increasing expression of endothelial cell adhesion factor-1 (ICAM-1) and fibrinogen activator inhibitor-1 (PAI-1) (Osada-Oka et al., 2016). Macrophage-derived exosomes play anti-inflammatory roles and regulate homeostasis in organisms (Mcdonald et al., 2014). Holder et al. reported that the human placenta takes up macrophage-derived exosomes in a time- and dose-dependent manner through clathrin-dependent endocytosis. Moreover, macrophage-derived exosomes induce the placenta to produce pro-inflammatory cytokines thus activating a response to maternal inflammation and infection and preventing damage to the fetus (Holder et al., 2016). Ye et al. reported that macrophage-derived exosomes are the main early secretors of pro-inflammatory cytokines in severe acute lung injury (ALI) and may activate neutrophils to produce several pro-inflammatory cytokines and IL-10. The IL-10 may then polarize macrophages to M2c, which may cause fibrosis after ALI (Ye et al., 2020).

### Other Diseases

**FIGURE 3:**
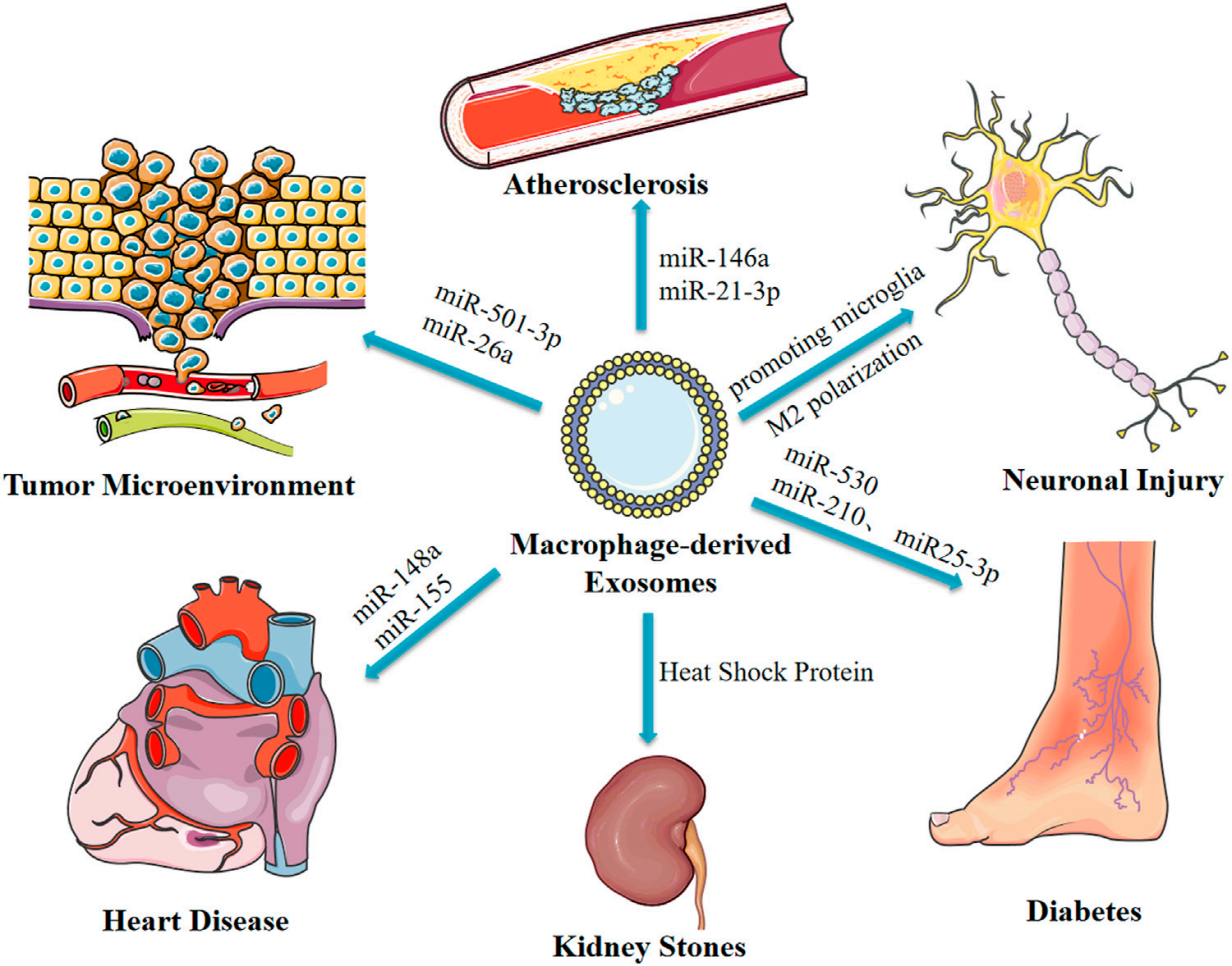
Macrophage-derived exosomes affect disease progression through delivery of miRNAs and other pathways.

Spasmolytic polypeptide-expressing metaplasia (SPEM) is the initial step of gastric precancerous lesions, which can progress to heterogeneous hyperplasia or even carcinoma with chronic inflammatory stimulation. Macrophages may be involved in this inflammatory process. Xu et al. reported that Deoxycholic acid-stimulated exosomes secreted by macrophages promote cellular communication between macrophages and gastric epithelial cells, thus facilitating development of SPEM, however, the study did not elucidate the mechanism (Xu et al., 2020). Macrophages are implicated in pathogenesis of kidney stones as they are involved in the immune response through the exosomal pathway after exposure to calcium hydroxalate (COM) crystal crystals. Although other proteins are involved, the main protein implicated in this process is the heat shock protein (Nilubon et al., 2018). LPS-stimulated exosomes secreted by macrophages inhibit neuronal inflammation in acute ischemia-induced neuronal injury by promoting microglia M2 polarization (Zheng et al., 2019). In addition, macrophage-derived exosomes from syphilis spirochete infection promote adhesion and permeability of human umbilical vein endothelial cells. Although the study did not explore the mechanisms underlying the activity, it reported that macrophage-derived exosomes are implicated in the pathogenesis of syphilis (Xu et al., 2019). These findings indicate the significance of macrophage-derived exosomes in development and progression several diseases. Therefore, these findings provide a basis for identification of new targets for treatment of different diseases (Figure 3).

## Applications of Macrophage-Derived Exosomes

### Engineering Macrophage-Derived Exosomes

Modification of exosomes through genetic or non-genetic approaches can enhance cytotoxicity and targeting of therapeutic agents, thus improving their effectiveness in killing cancer cells (Luan et al., 2017; You et al., 2018). Macrophage-derived exosomes can be packaged with various molecules to target tumor sites. Kim et al. developed and optimized a formulation of macrophage-derived exosome-loaded paclitaxel incorporating an aminoethylbenzamide-polyethylene glycol (AA-PEG) carrier fraction to target the overexpressed sigma receptor in lung cancer cells (Kim et al., 2018). The AA-PEG carrier exosome carrying PTX- (AA-PEG-exoPTX) exhibited a high drug loading capacity and high accumulation in cancer cells after systemic administration (Kim et al., 2018). Chemotherapy and surgery are the conventional treatments for triple-negative breast cancer (TNBC) due to a lack of effective therapeutic targets. However, limitations such as poor targeting and toxicity of chemotherapeutic agents limit the efficacy of chemotherapy and surgery. To circumvent this limitation, Li et al. developed a nano-delivery system of macrophage-derived exosome-encapsulated poly (lactic acid-hydroxyacetic acid) (Li et al., 2020a). The group modified the surface of the exosome with a peptide to target the mesenchymal-epithelial transition factor (c-Met), which is overexpressed by TNBC cells (Li et al., 2020a). A15 is the only ADAM protein containing an Arg-Gly-Asp (RGD) motif in its disintegrin-like domain. A15-rich exosomes with integrin αvβ3 increases affinity to tumor cells in an RGD-dependent manner (Chen et al., 2008; Ungerer et al., 2010). Gong et al. designed an A15-modified exosome-encapsulated adriamycin with a cholesterol-modified delivery system which exhibited synergistic anticancer effects *in vitro* and *in vivo* without adverse effects (Gong et al., 2019). Rayamajhi et al. combined macrophage-derived exosomes with synthetic liposomes and the bionic exosome improved the yield of exosomes, and improved targeting of tumor sites through encapsulation of adriamycin (Rayamajhi et al., 2019). These engineered macrophage-derived exosomes significantly improve targeting, however their safety should be evaluated (Luan et al., 2017).

### Application of Macrophage-Derived Exosomes as Drug Delivery Tools

In recent years, the use of exosomes as drug delivery systems has gained strong interest from researchers (Wei et al., 2017; Zhang et al., 2018). Previously, three cell-derived exosomes: pancreatic cancer cells (PCCs), pancreatic stellate cells (PSCs), and macrophages were used to deliver adriamycin. It was found that among the three types of exosomes, PCCs-derived exosomes had the highest drug loading efficiency whereas macrophage-derived exosomes loaded with adriamycin yielded the highest anti-tumor effect (Kanchanapally et al., 2019). Similarly, M1 macrophage-derived exosomes loaded with paclitaxel inhibited tumors by activating macrophage-mediated inflammation (Wang et al., 2019b). One factor that significantly limits the efficacy of chemotherapeutical medicine is multidrug resistance (MDR). Among the mechanisms that lead to MDR include overexpression of drug outflow transporter P-glycoprotein (Pgp) (Krishna et al., 2001; Sui et al., 2012). One study used ultrasound to encapsulate paclitaxel into exosomes (exoPTX) for delivery. The exoPTX inhibited the activity of p-gp thereby overcoming MDR in tumors. However, further investigations are needed to unravel the mechanism involved (Kim et al., 2016). Cisplatin is a platinum-containing anticancer drug that causes apoptosis primarily by damaging DNA and inhibiting replication and mitosis (Florea and Busselberg, 2011). A previous study (Zhang et al., 2020) found that umbilical cord-derived macrophages differentiated into M1 and M2 cells under the action of cytokines. M1 and M2 exosomes leaded with cisplatin fused with ovarian cancer cell line A2780 and cisplatin-resistant A2780/DDP leading to its accumulation in the cytoplasm near the nucleus, and reduce cisplatin IC50 (half maximal inhibitory concentration) of A2780 and A2780/DDP. Through this mechanism, it inhibits proliferation and promotes apoptosis of A2780 cells. In comparison, M1 exosomes loaded with cisplatin showed stronger anti-tumor effect than M2 exosomes. Nevertheless, molecules on the surface of exosomes that facilitate exosomal binding to target cancer cells should be further elucidated. In summary, macrophage-derived exosomes acquire macrophage properties such as the ability to target and modulate the tumor microenvironment and are therefore a promising vehicle for drug delivery. However, the application of exosomes as drug delivery systems is limited by low yields of exosomes from many tissues. This problem may be solved by constructing exosome-mimetic vesicles or genetic engineered exosomes (Xia et al., 2020).

### Use of Macrophage-Derived Exosomes as Gene and Protein Delivery Vehicles

**FIGURE 4:**
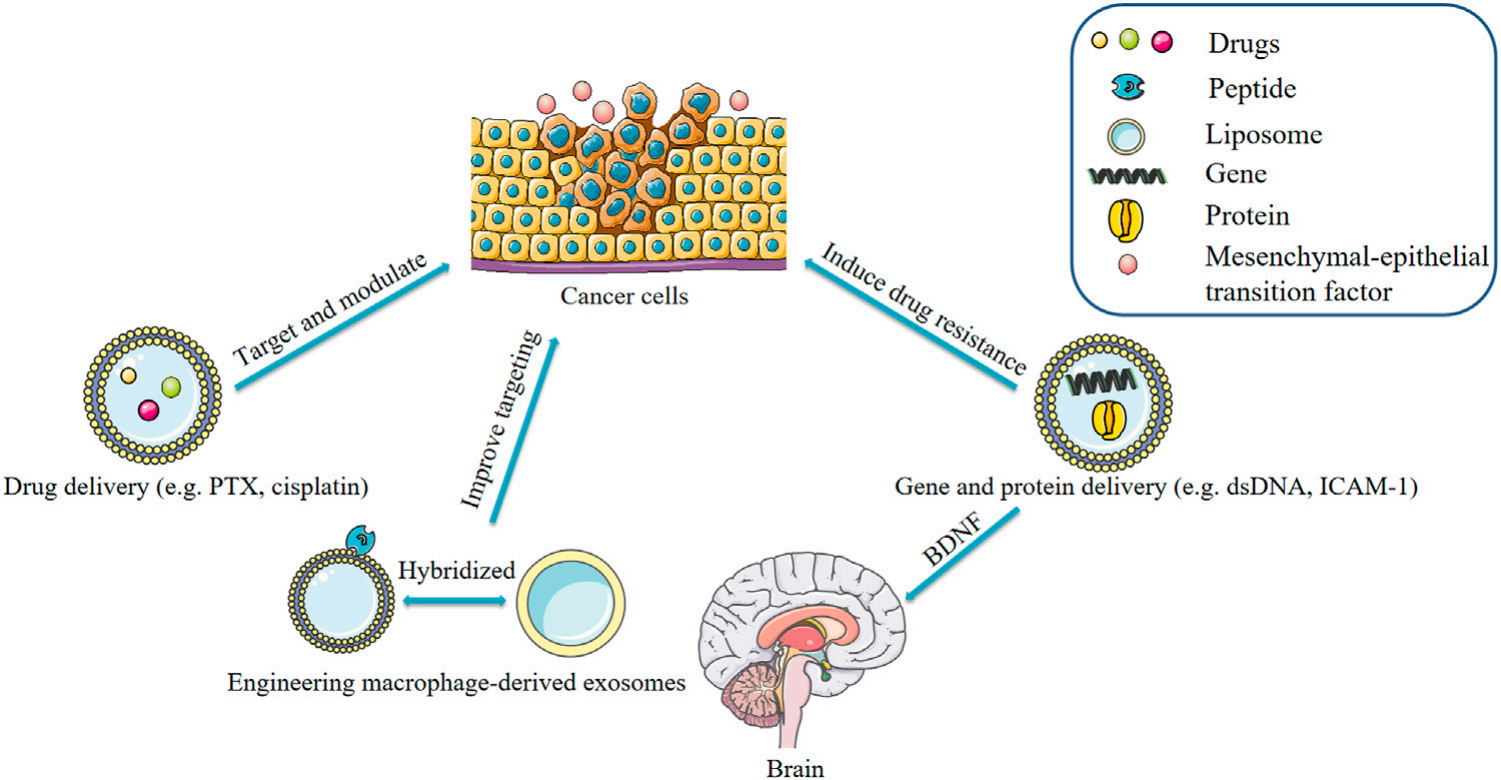
Applications of macrophage-derived exosomes.

Exosomes contain genes and proteins derived from parental cells. Recent research has found that macrophage-derived exosomes significantly decreased the sensitivity of PDAC (pancreatic ductal adenocarcinoma) cells to gemcitabine. In the study by Yoav et al., artificial dsDNA (barcode fragments) was transfected into mouse peritoneal macrophages and injected into mice bearing PDAC tumors. The concentration of barcode fragments was 4-fold higher in primary tumors and liver metastases than in normal tissue. This effect was mediated by the transfer of miR-365 in macrophage-derived exosomes. MiR-365 impaired activation of gemcitabine by upregulation of the triphospho-nucleotide pool in cancer cells and the induction of the enzyme cytidine deaminase; the latter inactivates gemcitabine (Yoav et al., 2018). Elsewhere, it was found that macrophage-derived exosomes contain integrin lymphocyte function-associated antigen 1 (LFA-1) acquired from parental cells (Yuan et al., 2017). These exosomes interact with intercellular adhesion molecule 1 (ICAM-1) and transport a brain-derived neurotrophic factor (BDNF) to the brain. TAMs are characterized by M2-polarized phenotype and have been shown to promote the migration of gastric cancer cells. A recent study suggested that M2 macrophage-derived exosomes mediate an intercellular transfer of ApoE-activating PI3K-Akt signaling pathway in recipient gastric cancer cells to remodel the cytoskeleton-supporting migration. Because ApoE is a highly specific and effective protein in M2 macrophages-derived exosomes. Of note, exosomes derived from M2 macrophages of ApoE^−/−^ mice did not affect the migration of gastric cancer cells (Zheng et al., 2018). The delivery of a therapeutic miRNA or protein to its target tissue or cell has been a challenging task (Zhang et al., 2018). Recent studies have confirmed that macrophage-derived exosomes have great potential in the treatment of diseases by serving as delivery vehicles for genes and proteins. It is possible to modify parental cells and use transgenesis to make such cells secrete exosomes containing the desired therapeutic protein (Figure 4).

## Conclusion and Perspectives

In summary, macrophage-derived exosomes have important role in the treatment of diseases such as tumors, atherosclerosis, and diabetes. When used as delivery vehicles, they bind to receptors on target cells thereby delivering loaded drugs such as proteins and nucleic acids. The function of macrophage-derived exosomes is influenced by macrophage polarization, which regulated by the surrounding inflammatory environment. Inflammation is usually an alternating process, thus the role of macrophage-derived exosomes should be viewed in a dynamic light.

Compared to artificially targeted nanocarriers, macrophage-derived exosomes are safer and can be easily modified for application in gene therapy. The following issues also need to be resolved in future studies: 1) Currently, there is no uniform protocol to isolate, purify, and preserve exosomes. Moreover, some of the existing isolation and purification methods are not effective. 2) The low yields of exosomes from specific donor cells limits their application as targeted drug carrier systems. 3) There are few pharmacokinetic studies on the use of macrophage-derived exosomes as drug delivery tools, which limits their development as biopharmaceuticals. It is believed that with the development of biotechnology, macrophage-derived exosomes will play a key role in the diagnosis, prevention, and treatment of diseases in the future.
